# Complex poisoning mainly with benzyl alcohol complicated by paralytic ileus: a case report

**DOI:** 10.1186/s12245-022-00434-4

**Published:** 2022-07-04

**Authors:** Hirotsugu Fukuda, Ryo Kamidani, Hideshi Okada, Yuichiro Kitagawa, Takahiro Yoshida, Shozo Yoshida, Shinji Ogura

**Affiliations:** 1 Department of Emergency and Disaster Medicine, Gifu University Graduate School of Medicine, Gifu, 501-1194 Japan; 2 Abuse Prevention Center, Gifu University Graduate School of Medicine, Gifu, Japan

**Keywords:** Benzyl alcohol poisoning, Paralytic ileus, Poisoning, Hydrogen peroxide, Case report

## Abstract

**Background:**

Benzyl alcohol is used as stripping agent in paints and other applications, and benzyl alcohol poisoning is indicated by symptoms, such as impaired consciousness, respiratory depression, hypotension, metabolic acidosis, and renal dysfunction.

**Case presentation:**

A 27-year-old Asian man was transported to a hospital for severe disturbance of consciousness following exposure to a paint stripper containing benzyl alcohol, ethylene glycol, and hydrogen peroxide, which he was using to repaint a bridge. The patient was treated under sedation for benzyl alcohol poisoning. On day 3 of hospitalization, his abdominal computed tomography scan revealed a paralytic ileus, so he was transferred to our hospital. The combined toxicity from multiple substances, mainly benzyl alcohol, was thought to be a contributing factor for the paralytic ileus. Upon arrival, the patient also had chemical burns, hypernatremia, and elevated myogenic enzyme levels. His urinary hippuric acid level was high (14.9 g/L) upon admission to the previous hospital. We treated the patient with artificial respiration management, while avoiding high-density oxygen, and with gastrointestinal decompression by gastric tube implantation; laxatives were also administered. The paralytic ileus improved on the 4th day, the tube was removed on the 6th day, and the patient was discharged on the 11th day of hospitalization. No apparent complications were observed at discharge.

**Conclusions:**

To the best of our knowledge, this is the first case report of paralytic ileus caused by benzyl alcohol, although multiple factors may have influenced the symptoms. After exposure to benzyl alcohol by inhalation and dermal absorption, the patient developed impaired consciousness, metabolic acidosis, and paralytic ileus, and the presence of elevated urinary hippuric acid led to a definitive diagnosis.

## Background

Benzyl alcohol is used as a stripping agent in paints and other industrial applications and even found in cosmetic products and food additives. Benzyl alcohol is a clear, colorless liquid that is readily available commercially. Although it was previously used as an additive in injectable drugs, its use has been discontinued because of problems, such as “gasping syndrome” in infants [1–3].

There are very few reports on benzyl alcohol poisoning in adults, and to our knowledge, there are no reports of poisoning complicated with paralytic ileus. Here, we report the case of a patient with impaired consciousness and paralytic ileus due to complex poisoning mainly from benzyl alcohol.

## Case presentation

A 27-year-old Asian man was transported to a nearby general hospital because he experienced severe disturbance of consciousness while using painting material stripper containing benzyl alcohol, ethylene glycol, and hydrogen peroxide to repaint a bridge in the summer. There was no relevant medical history of laparotomy and no noteworthy family history or psychosocial history of addiction.

On arrival at the nearby general hospital, his Glasgow Coma Scale score was 3 (eye, 1; verbal, 1; motor, 1), with no fever and elevated white blood cell count of 25.7 × 10^3^/μL. Computed tomography (CT) and magnetic resonance imaging (MRI) scans of the head showed no abnormal findings. Based on field conditions and interviews with the workplace supervisor, benzyl alcohol poisoning was suspected. The patient was restless and managed with midazolam under sedation, supplemental fluids, and oxygen administration. On the second day of admission, the results of his blood tests revealed worsening of liver and kidney function and metabolic acidosis (Table 1). Since paralytic ileus was suspected on the third day of admission, the patient was transferred to our hospital for further investigation and treatment. No specific treatment for poisoning or ileus paralysis was administered by the previous physician.

**Table 1:** Laboratory findings on day 2 after admission to the previous hospital

< Complete blood count **> (normal range)**					
White blood cells	44.2 × 10^3^ (3.5–9.1 × 10^3^)	/uL	Sodium	136 (136–147)	mmol/L
Red blood cells	5.44 × 10^6^ (3.7–5.0 × 10^6^)	/uL	Potassium	7.6 (3.6–5.0)	mmol/L
Hemoglobin	17.4 (11.3–15.2)	g/dL	Chloride	103 (98–109)	mmol/L
Hematocrit	47.6 (40–48)	%	C-reactive protein	80,700 (≤ 3000)	μg/L
Platelet	329 × 10^4^ (130–369 × 10^4^)	/uL			
			**< Arterial blood gas > (normal range)**		
**< Biochemistry > (normal range)**			FiO_2_	0.21	
Total protein	7.4 (6.7–8.3)	g/dL	pH	7.278 (7.38–7.46)	
Albumin	4.6 (3.8–5.2)	g/dL	PaCO_2_	19.8 (32–46)	mmHg
Creatinine kinase	518 (62–287)	IU/L	PaO_2_	63.9 (74–108)	mmHg
Aspartate transaminase	56 (10–40)	IU/L	HCO_3−_	9.0 (21–29)	mmol/L
Alanine transaminase	35 (5–40)	IU/L	Base excess	−14.9 (−2 to 2)	mmol/L
Lactate dehydrogenase	854 (115–359)	IU/L			
Alkaline phosphatase	356 (115–359)	IU/L			
γ-GTP	52 (≤ 70)	IU/L			
Creatinine	237.8 (41.5–69.8)	μmol/L			
Blood urea nitrogen	8.2 (2.8–7.8)	mmol/L			

*γ-GTP* γ-glutamyl transpeptidase, *F*_*I*_*O*_*2*_ Fraction of inspiratory oxygen, *PaCO*_*2*_ Partial pressure of arterial carbon dioxide, *PaO*_*2*_ Partial pressure of arterial oxygen, *HCO*_*3*−_ Bicarbonate

**Fig. 1:**
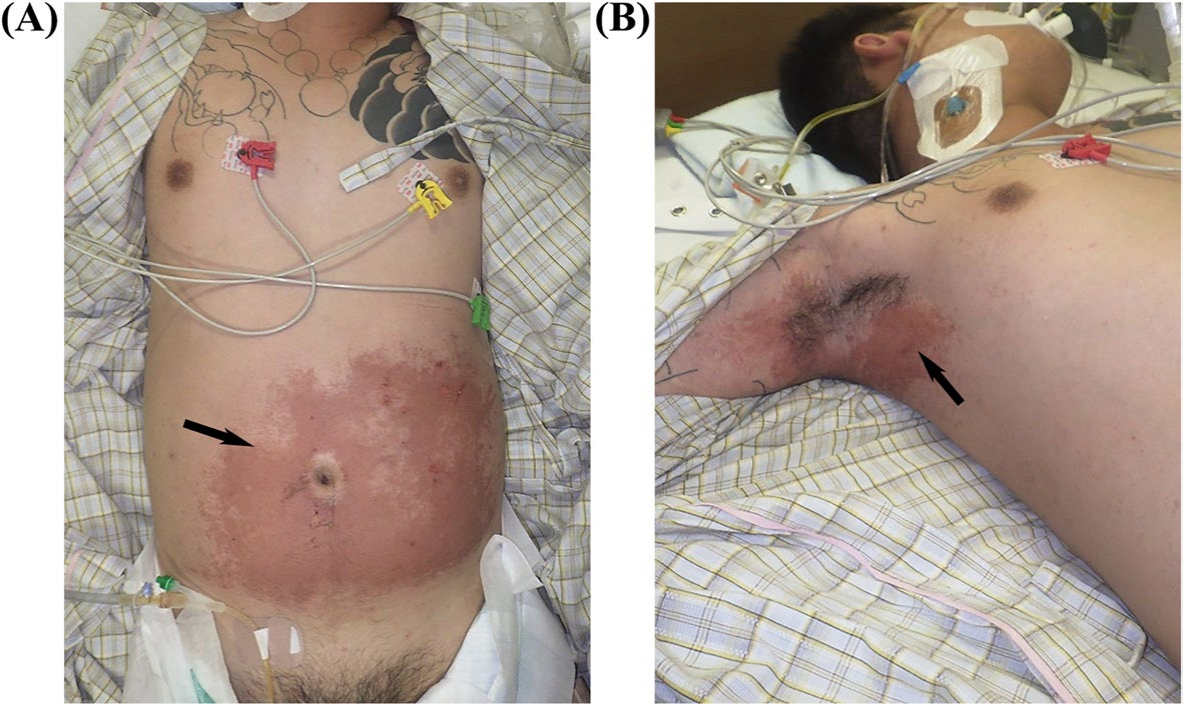
Skin findings on admission. Blister caps and epidermolysis were found on the abdomen, axillae, elbows, buttocks, and right knee

On arrival at our hospital, the patient’s Glasgow Coma Scale score was 9 (eye, 2; verbal, 3; motor, 4); he was administered midazolam under sedation (intravenous drip infusion; 5 mg/h). On physical examination, body temperature of 37.7 °C, respiratory rate of 20 breaths/min with a normal respiratory pattern, heart rate of 84 beats/min, and blood pressure of 151/90 mmHg were observed. Auscultation revealed no abnormalities in the heart and lung sounds. Saturation of percutaneous oxygen was measured at 98% in room air, and arterial blood gas analysis showed a partial pressure of arterial oxygen (PaO_2_) of 87.3 mmHg and alveolar-arterial oxygen difference of 23.2 mmHg; his quick sequential organ failure assessment score was 1. His abdomen was distended, and bowel sounds were attenuated. Blister caps and epidermolysis were found on the abdomen, axillae, elbows, buttocks, and right knee (Fig. 1). The total body surface area, burn index, and prognostic burn index were 14%, 7%, and 34%, respectively.

His laboratory test results showed hypercoagulability (levels of fibrinogen, 733 mg/dL; fibrin/fibrinogen degradation product, 8.4 μg/mL; and D-dimer, 40 μg/mL), elevated levels of myogenic deviation enzymes (creatinine kinase, 1803 IU/L; aspartate transaminase, 64 IU/L; and lactate dehydrogenase, 392 IU/L), and hypernatremia (Na; 148 mmol/L) (Table 2). Urinary screening was performed using a simple drug screening kit, Trigae®DOA (Sysmex Corporation, Kobe, Japan), which showed positive results for benzodiazepines under continuous intravenous midazolam administration and negative results for other drugs, including opioids.

**Table 2:** Laboratory findings at the time of admission

< Complete blood count **> (normal range)**			**< Coagulation status > (normal range)**		
White blood cells	6,600 (3.5–9.1 × 10^3^)	/uL	APTT	30.3 (24.3–36.0)	s
Red blood cells	4.7 × 10^6^ (3.7–5.0 × 10^6^)	/uL	PT-INR	0.95 (0.85–1.15)	
Hemoglobin	12.0 (11.3–15.2)	g/dL	Fibrinogen	733 (150–400)	mg/dL
Hematocrit	42.7 (40–48)	%	FDP	8.4 (≤ 4.0)	μg/mL
Platelet	227 × 10^4^ (130–369 × 10^4^)	/uL	D-dimer	40 (< 1,000)	μg/mL
**< Biochemistry > (normal range)**			**< Arterial blood gas > (normal range)**		
Total protein	6.4 (6.7–8.3)	g/dL	F_I_O_2_	0.21	
Albumin	3.6 (3.8–5.2)	g/dL	pH	7.413 (7.38–7.46)	
Creatinine kinase	1,803 (62–287)	IU/L	PaCO_2_	33.9 (32–46)	mmHg
Aspartate transaminase	64 (10–40)	IU/L	PaO_2_	87.3 (74–108)	mmHg
Alanine transaminase	62 (5–40)	IU/L	HCO_3_^-^	21.2 (21–29)	mmol/L
Lactate dehydrogenase	392 (115–359)	IU/L	Base excess	-2.1 (-2 to 2)	mmol/L
Alkaline phosphatase	236 (115–359)	IU/L	Lactate	5.4 (0.3–1.9)	mmol/L
γ-GTP	35 (≤ 70)	IU/L	Anion gap	12.1 (11.8–12.2)	mmol/L
ChE	327 (242–495)	IU/L			
Amylase	254 (37–125)	IU/L	**< Urinalysis > (normal range)**		
Creatinine	72.5 (41.5–69.8)	μmol/L	Urinary—sodium	11 (50–300)	mmol/L
Blood urea nitrogen	10.6 (2.8–7.8)	mmol/L	Urinary—potassium	24.1 (12–130)	mmol/L
Triglyceride	2.80 (0.56–1.68)	mmol/L	Urinary—chloride	75 (50–330)	mmol/L
Total cholesterol	4.37 (3.88–5.66)	mmol/L	Urinary—urea nitrogen	1847 (10–15)	mmol/L
Sodium	148 (136–147)	mmol/L	Urinary sugar	113 (≤ 20)	mmol/L
Potassium	4.0 (3.6–5.0)	mmol/L	Urinary osmolality	714 (581–1136)	mOsm/kgH_2_O
Chloride	118 (98–109)	mmol/L	Urinary hippuric acid	0.63 (< 1.0)	g/L
Blood sugar	12.2 (3.9–6.1)	mmol/L			
HbA1c	5.5 (4.6–6.2)	%			
C-reactive protein	41,100 (≤ 3000)	μg/L			

HbA1c is expressed as National Glycohemoglobin Standardization Program (NGSP) values. *γ-GTP*, γ-glutamyl transpeptidase; *ChE* Cholinesterase, *APTT* Activated partial thromboplastin time, *PT-INR* Prothrombin time-international normalized ratio, *FDP* Fibrin/fibrinogen degradation products, *F*_*I*_*O*_*2*_ Fraction of inspiratory oxygen, *PaCO*_*2*_ Partial pressure of arterial carbon dioxide, *PaO*_*2*_ Partial pressure of arterial oxygen, *HCO*_*3*−_ Bicarbonate

**Fig. 2:**
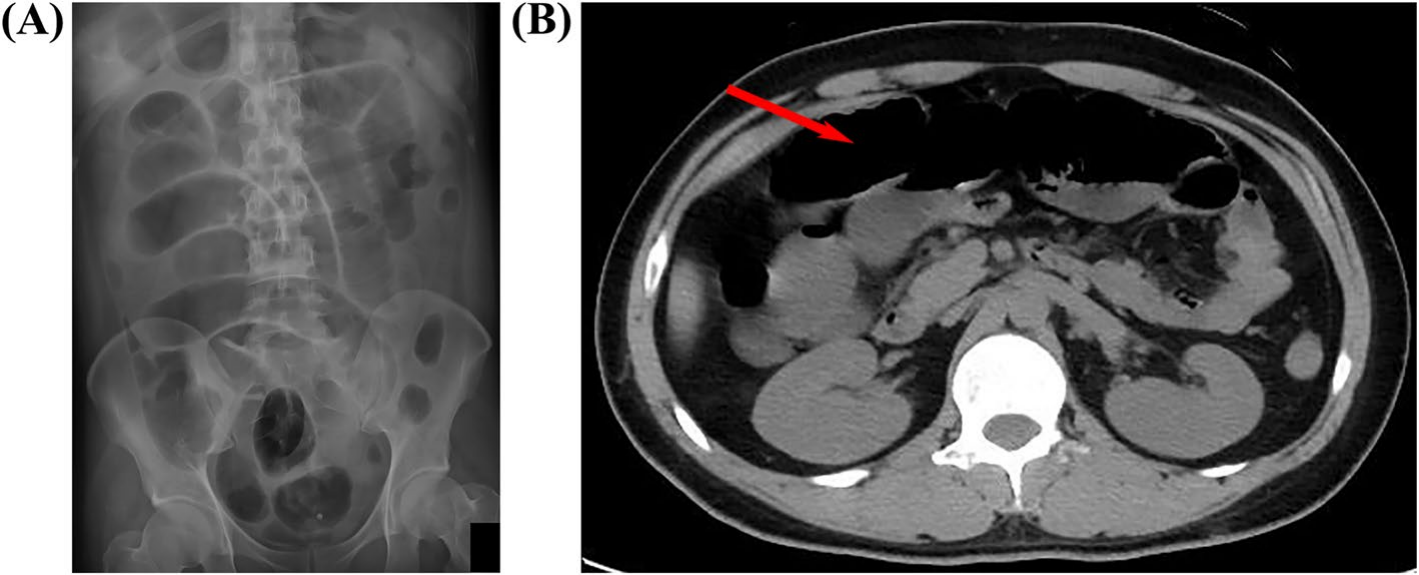
Abdominal radiograph and CT on admission. **A** Abdominal radiograph revealed intestinal gas and dilatation of the intestine.**B** Abdominal CT showed dilatation (red arrow) and fluid retention in the small intestine

His abdominal radiographic scan showed dilatation of the intestine (Fig. 2A), and abdominal CT scan showed no obvious obstruction, although small bowel dilatation and fluid retention were observed (Fig. 2B). No other organs showed abnormal findings.

**Fig. 3:**
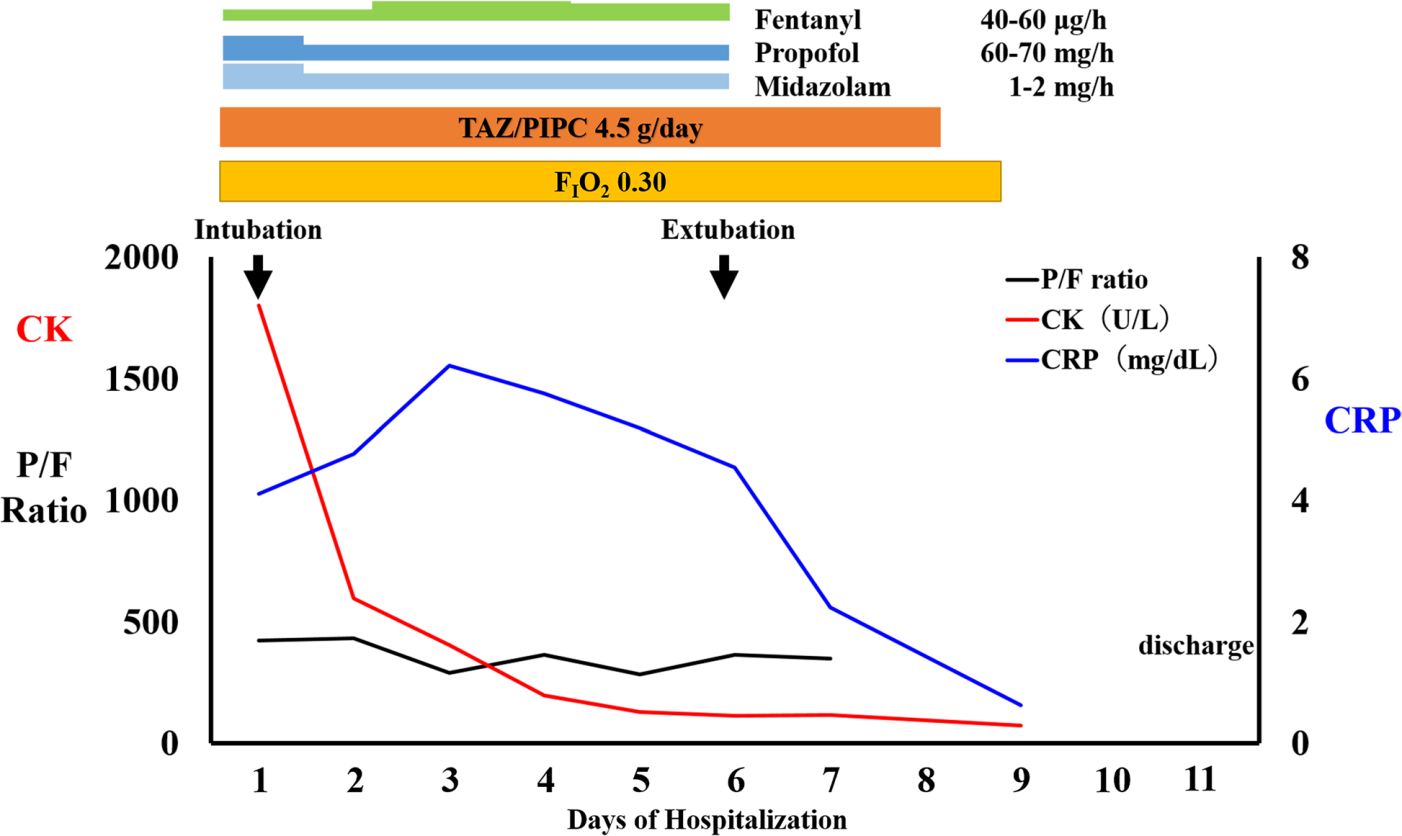
Clinical course. P/F ratio, PaO_2_/F_I_O_2_ ratio; CK, creatinine kinase; CRP, C-reactive protein; TAZ/PIPC, tazobactam/piperacillin; F_I_O_2_, fraction of inspiratory oxygen; PaO_2_, partial pressure of arterial oxygen

As the patient was in the acute stage when he was transferred to our hospital, symptomatic treatment was considered the only feasible treatment option for the poisoning; hence, the patient was intubated and managed with an inhaled oxygen concentration of 30% (Fig. 3). Next, a gastric tube was placed, and laxatives were administered for the paralytic ileus; furthermore, he was started on tazobactam/piperacillin considering the risk of bacterial translocation or generalized peritonitis. Additionally, we treated his metabolic acidosis with infusion crystalloids and skin injury with daily washing and ointment application. On the second day after the patient was transferred to our hospital, a high level of urinary hippuric acid (14.9 g/dL; reference range, < 1.0 g/L) was observed, consistent with the finding of benzyl alcohol poisoning.

**Fig. 4:**
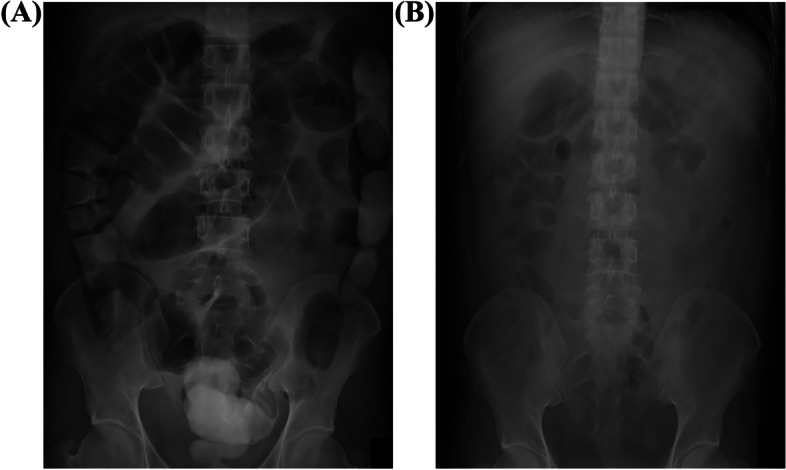
Changes in abdominal radiographs after treatment. Abdominal radiographs on admission (**A**) and hospital day 6 (**B**)

After transfer to our hospital, the patient’s progress was good, his laboratory data gradually improved, and his PaO_2_/fraction of inspired oxygen (F_I_O_2_) did not worsen. On the fourth day at our hospital, contrast medium was administered through the gastric tube, and on the fifth day, it had reached the rectum; hence, tube feeding was initiated (Fig. 4). On the sixth day, bowel movement was confirmed, so the patient was extubated, and oral feeding was started. No complications were observed, and the patient was discharged on the tenth day.

## Discussion

We encountered a patient with impaired consciousness and a paralytic ileus due to complex benzyl alcohol poisoning. To the best of our knowledge, this is the first case report of paralytic ileus after exposure to benzyl alcohol. The patient developed impaired consciousness, metabolic acidosis, and paralytic ileus, and the presence of elevated urinary hippuric acid led to a definitive diagnosis. It is important to be aware of the possibility of exposure to multiple poisonous substances via multiple routes, especially in enclosed spaces during the summer.

Benzyl alcohol is a key ingredient in many paint removers and can be absorbed orally, by inhalation, or transdermally. It is oxidized by alcohol-depleting enzymes in the liver to produce benzoic acid, which is then combined with glycine and excreted in urine as hippuric acid [4]. Benzyl alcohol poisoning in humans causes impaired consciousness, respiratory depression, hypotension, metabolic acidosis, renal dysfunction, and hypothermia. Laboratory findings of benzyl alcohol poisoning include anion gap-opening metabolic acidosis, high creatinine kinase level, and hypernatremia [4]. It is thought that benzyl alcohol causes direct injury to the central nervous system, but the evidence is lacking [1].

There are several reports of “gasping syndrome” in premature infants in the past, where the symptoms appeared when the blood concentration of benzyl alcohol increased after continuous administration of antimicrobials or after umbilical catheter cleaning [1–3, 5]. Reports of acidosis in children and adults are quite rare; Lopez-Herce et al. reported a case of hypotension and high anion gap-opening metabolic acidosis caused by benzyl alcohol added to continuous intravenous diazepam infusion in a 5-year-old girl with seizure aggravation [6]. Smith et al. reported a case of convulsions and respiratory arrest in an adult after intravenous administration of 9000 mg benzyl alcohol, which was added as a preservative to etoposide, during chemotherapy for malignant lymphoma [7]. Another report highlighted a case of an adult intoxicated patient with symptoms of sudden altered mental status, headache, anorexia, metabolic acidosis, hypokalemia, hypophosphatemia, and hyperammonemia associated with renal tubular dysfunction due to inhalation exposure to benzyl alcohol. As mentioned above, the condition is currently characterized by a wide variety of symptoms [8]. In the present case, we postulated that the consciousness disorder and metabolic acidosis were induced by benzyl alcohol because the patient did not have a high body temperature when he was admitted to the previous hospital, so heatstroke could be ruled out, and there was no notable history of illness. However, since hippuric acid is a metabolite of toluene, an organic solvent, inhaled toluene is also excreted in the urine as hippuric acid. Therefore, in this case, the patient was diagnosed with benzyl alcohol poisoning from the components of the exfoliant he used, although toluene poisoning is a differential diagnosis of benzyl alcohol poisoning [4].

However, there is no specific treatment, so systemic management must be performed symptomatically [4]. In the present case, the patient also had a paralytic ileus. This patient had no notable medical history or history of opioid use before onset, there was no active suspicion of infection based on clinical data and findings, and there was no other known cause of paralytic ileus. Therefore, the possibility of poisoning with other ingredients must be considered. The main ingredients of the paint removal agent that the patient had used were benzyl alcohol, hydrogen peroxide, and ethylene glycol. It has been suggested that hydrogen peroxide can cause oxygen gas embolism in the blood vessels due to the decomposition of hydrogen peroxide into oxygen in the bloodstream [9, 10]. However, hydrogen peroxide breaks down quickly in the blood and is a very small molecule, so confirming the diagnosis of hydrogen peroxide poisoning is difficult [11]. In addition, there was no perioral or oral damage in this case, and ethylene glycol is considered to have poor percutaneous absorption. However, we speculated that hydrogen peroxide, ethylene glycol, and other substances, including unknown substances not detected in routine medical practice, combined with benzyl alcohol may have produced a complex toxic effect leading to the development of paralytic ileus and other symptoms. Therefore, since the involvement of hydrogen peroxide could not be ruled out, we maintained F_I_O_2_ levels of around 0.3 and avoided high concentrations of oxygen to suppress the formation of free radicals and promote oxygen dissolution.

In cases of industrial product poisoning, there is a possibility of exposure to multiple toxic substances in addition to the main substance, and unexpected symptoms may occur. The present case highlights that benzyl alcohol poisoning should be suspected in patients with paralytic ileus, sudden altered mental status, and metabolic acidosis.

## Data Availability

The datasets used and/or analyzed during the current study are available from the corresponding author upon reasonable request.

## Abbreviations

CT: Computed tomography
MRI: Magnetic resonance imaging
F_I_O_2_: Fraction of inspired oxygen
PaO_2_: Partial pressure of arterial oxygen
